# Mechanisms Leading to Rhythm Cessation in the Respiratory PreBötzinger Complex Due to Piecewise Cumulative Neuronal Deletions^1^,^2^,^3^

**DOI:** 10.1523/ENEURO.0031-15.2015

**Published:** 2015-08-31

**Authors:** Hanbing Song, John A. Hayes, Nikolas C. Vann, M. Drew LaMar, Christopher A. Del Negro

**Affiliations:** 1 Department of Applied Science, The College of William & Mary, Williamsburg, Virginia 23187-8795; 2 Department of Biology, The College of William & Mary, Williamsburg, Virginia 23187-8795

**Keywords:** cumulative ablation, Dbx1, network, recurrent excitation, rhythmogenesis, synaptic transmission

## Abstract

The mammalian breathing rhythm putatively originates from Dbx1-derived interneurons in the preBötzinger complex (preBötC) of the ventral medulla. Cumulative deletion of ∼15% of Dbx1 preBötC neurons in an *in vitro* breathing model stops rhythmic bursts of respiratory-related motor output. Here we assemble *in silico* models of preBötC networks using random graphs for structure, and ordinary differential equations for dynamics, to examine the mechanisms responsible for the loss of spontaneous respiratory rhythm and motor output measured experimentally *in vitro*. Model networks subjected to cellular ablations similarly discontinue functionality. However, our analyses indicate that model preBötC networks remain topologically intact even after rhythm cessation, suggesting that dynamics coupled with structural properties of the underlying network are responsible for rhythm cessation. Simulations show that cumulative cellular ablations diminish the number of neurons that can be recruited to spike per unit time. When the recruitment rate drops below 1 neuron/ms the network stops spontaneous rhythmic activity. Neurons that play pre-eminent roles in rhythmogenesis include those that commence spiking during the quiescent phase between respiratory bursts and those with a high number of incoming synapses, which both play key roles in recruitment, i.e., recurrent excitation leading to network bursts. Selectively ablating neurons with many incoming synapses impairs recurrent excitation and stops spontaneous rhythmic activity and motor output with lower ablation tallies compared with random deletions. This study provides a theoretical framework for the operating mechanism of mammalian central pattern generator networks and their susceptibility to loss-of-function in the case of disease or neurodegeneration.

## Significance Statement

Interneurons of the preBötzinger complex (preBötC) play an essential role in respiratory rhythmogenesis in rodents, which may apply to mammals in general, including humans. Piecewise destruction of the preBötC, which retains spontaneous function *in vitro*, impairs rhythmicity and eventually irreversibly terminates respiratory rhythmogenesis. However, little is known regarding the reason for rhythm cessation. The present study shows that basic network structure is retained, but nevertheless cumulative neuron deletions disturb the process of recurrent excitation, leading to the failure of burst initiation and the loss of spontaneous rhythmic activity. This study proposes a plausible explanation for how the respiratory oscillator loses functionality when subject to piecewise destruction, which could help explain neurodegenerative disease etiology.

## Introduction

Inspiratory breathing movements originate within the preBötzinger complex (preBötC) of the lower medulla (Smith et al., 1991; Feldman and Del Negro, 2006; Feldman et al., 2013; Moore et al., 2013). The underlying mechanisms that generate inspiratory rhythm and their susceptibility to failure in conditions of deterioration or disease remain incompletely understood. Here we address these interrelated issues using modeling and simulation.

Interneurons whose progenitors express the homeodomain transcription factor Dbx1 (i.e., Dbx1 neurons) are glutamatergic and form local and commissural excitatory synaptic connections. These neurons may comprise the excitatory rhythmogenic core of the preBötC according to the Dbx1 core hypothesis (Bouvier et al., 2010; Gray et al., 2010; Picardo et al., 2013), which predicts that cumulative destruction of Dbx1 neurons in the preBötC should irreversibly impair and then prohibit respiratory rhythm by degrading the core oscillator. This prediction was experimentally tested by photonically destroying Dbx1 preBötC neurons in rhythmically active slices that retain the preBötC, while monitoring breathing-related motor output from the hypoglossal (XII) cranial nerve. Inspiratory motor output decreased in amplitude and frequency until the spontaneous rhythm irreversibly terminated after ∼15% of the Dbx1 preBötC population was destroyed (Wang et al., 2014).

These data support the Dbx1 core hypothesis but cannot explain the cessation of network rhythm, which could have structural or dynamical explanations. First, loss of rhythm could coincide with a precipitous drop in network connectivity (i.e., structure), which impairs neuronal communication and thus precludes coordinated activity. Second, removing constituent neurons could decrease excitability or lower baseline membrane potential of the neurons that remain and thus impede burst generation. It is also possible that cumulative cellular ablations cause structural and dynamical changes whose combined effects halt rhythmic activity.

Inspiratory neural bursts in the preBötC depend on excitatory synaptic transmission (Greer et al., 1991; Funk et al., 1993; Ge and Feldman, 1998; Rekling et al., 2000; Wallén-Mackenzie et al., 2006). According to the logic of a canonical network oscillator (Grillner, 2006) recurrent excitation spreads from active presynaptic neurons to quiescent postsynaptic partners, which causes temporal summation and recruitment to the active-burst phase (Rubin et al., 2009). Cumulatively ablating ∼39 neurons in the model preBötC (330 neurons in total) stops spontaneous rhythmic function. Here we show that this loss-of-function is not linked solely to deterioration of network structure as determined by a subset of formal network measures, but by the combined effect of loss of network structure and neuronal dynamics. Selectively targeting neurons that have a large number of incoming synapses decreases the ablation tally considerably, emphasizing the importance of these synaptic properties for spontaneous rhythmic function. We conclude that rhythm cessation is attributable to the loss of constituent neurons with large numbers of incoming synapses or high excitability (or both), which impedes recurrent excitation by diminishing the rate at which synaptic transmission among network constituents recruits more neurons to join the active phase. Failing to reach a threshold rate of 1 neuron/ms, recurrent excitation cannot spread fast enough to recruit the entire network; subsequent spontaneous bursts no longer occur. This study is important for understanding basic mechanisms of rhythm generation and potentially for restoring functionality to arrhythmic networks in pathological conditions and disease.

## Materials and Methods

### Rubin–Hayes preBötC interneuron model

Each node (i.e., neuron) is populated with a Rubin–Hayes preBötC model (Rubin et al., 2009; Dunmyre et al., 2011), which features Hodgkin–Huxley-like spiking currents and four additional currents: calcium-activated nonspecific cation current (*I*_CAN_; Crowder et al., 2007; Pace et al., 2007b; Mironov, 2008, 2013; Pace and Del Negro, 2008; Mironov and Skorova, 2011), persistent sodium current (*I*_Na-P_; Del Negro et al., 2002a; Ptak et al., 2005; Koizumi and Smith, 2008), excitatory synaptic current mediated predominantly by AMPA receptors (*I*_syn_; Funk et al., 1993; Ge and Feldman, 1998; Wallén-Mackenzie et al., 2006), and electrogenic Na/K ATPase pump current (*I*_pump_) (Del Negro et al., 2009; Krey et al., 2010). The model features material balance equations for intracellular calcium and sodium concentrations. The Rubin–Hayes model is in the public domain (http://senselab.med.yale.edu/modeldb/ShowModel.asp?model=125649).

The current-balance equation takes the form:

$$\frac{dV}{dt}=-I_{leak}\left(V\right)-I_{Na}\left(V,m,h\right)-I_{k}\left(V,n\right)-I_{CAN}\left(V,Ca\right)-I_{Na-P}\left(V,h_{Na-P}\right)-I_{syn}\left(V,s_{1}\ldots s_{N}\right)-I_{pump}(Na),$$

where

$$\frac{dx}{dt}=\left(x_{\infty }\left(V\right)-x\right)/T_{x}(V)$$

$$\frac{ds}{dt}=\left(\left(1-s\right)s_{\infty }\left(V\right)-k_{s}s\right)/T_{s}$$

$$\frac{dCa}{dt}=\varepsilon \left(\overset{N}{\underset{i=1}{\sum }}s_{i}\;\cdot \;k_{IP3}-k_{Ca}\left(Ca-Ca_{\infty }\right)\right)$$

$$\frac{dNa}{dt}=\alpha \left(-I_{CAN}\left(V,Ca\right)-I_{pump}\left(Na\right)\right)$$

describe the evolution of the state variables, for each $x\;\in \;\{m,h,n,h_{Na-P}\}$.

Membrane currents are described with chord-conductance equations, in some cases modified for Ca^2+^ or Na^+^ gating (*I*_CAN_ and *I*_pump_; Li et al., 1996):

$I_{leak}\left(V\right)=g_{leak}\left(V-E_{L}\right)$
$I_{Na}\left(V,m,h\right)=g_{Na}m^{3}h\left(V-E_{Na}\right)$
$I_{Na-P}\left(V,h_{Na-P}\right)=g_{Na-P}m_{Na-P_{\infty }}h_{Na-P}\left(V-E_{Na}\right)$
$I_{K}\left(V,n\right)=g_{K}n^{4}\left(V-E_{K}\right)$
$I_{CAN}\left(V,Ca\right)=g_{CAN}\left(V-E_{CAN}\right)/(1+\exp\left(\left(Ca-k_{CAN}\right)/\sigma_{CAN}\right))$
$I_{syn}\left(V,s_{1}\;\ldots \;s_{N}\right)=g_{syn}\overset{N}{\underset{i=1}{\sum }}s_{i}(V-E_{syn})$,

where *N* is the number of presynaptic neurons and $\left\{s_{1}\ldots s_{N}\right\}$ is the set of presynaptic *s* variables, and $I_{pump}\left(Na\right)=r_{pump}(\phi \left(Na\right)-\phi \left(Na_{\infty }\right))$.

The remaining functions in the model, including those representing the voltage-dependence of channel kinetics, are as follows:

$$x_{\infty }\left(V\right)=1/\left(1+\exp\left(\frac{V-\theta_{x}}{\sigma_{x}}\right)\right)$$

$$T_{x}\left(V\right)=T_{x_{max}}/\cosh\left(\frac{V-\theta_{x}}{2\sigma_{x}}\right)$$

$$\phi \left(Na\right)=Na^{3}/\left(Na^{3}+k_{Na}^{3}\right)$$

Model parameters are set to the following values, unless otherwise specified:

$$C=45\;pF,g_{leak}=3\pm 0.78\;nS,E_{L}=-61.46\;mV,g_{Na}=150\;nS,g_{Na-P}=1\;nS,g_{K}=30\;nS,$$

$$E_{K}=-75\;mV,g_{CAN}=4\pm 0.75\;nS,E_{CAN}=0\;mV,g_{syn}=3.25\;nS,E_{syn}=0\;mV,$$

$$\theta_{m}=-36\;mV,\sigma_{m}=-8.5\;mV,T_{m_{\max}}=1\;ms,\theta_{h}=-30\;mV,\sigma_{h}=5\;mV,T_{h_{\max}}=15\;ms,$$

$$\theta_{n}=-30\;mV,\sigma_{n}=-5\;mV,T_{n_{\max}}=30\;ms,\theta_{s}=15\;mV,\sigma_{s}=-3\;mV,T_{s}=15\;ms,$$

$$\theta_{m_{Na-P}}=-40\;mV,\sigma_{m_{Na-P}}=-6\;mV,\theta_{h_{Na-P}}=-48\;mV,\sigma_{h_{Na-P}}=6\;mV,$$

$$T_{Na-P}=15ms,k_{Ca}=22.5\;ms^{-1},k_{CAN}=0.9,\sigma_{CAN}=-0.05\;mV,k_{s}=1,$$

$$k_{IP3}=1200\;\mu M\cdot ms^{-1},r_{pump}=200\;pA,k_{Na}=10\;mM,Ca_{\infty }=0.05\;\mu M,Na_{\infty }=5\;mM,$$

$$\varepsilon =0.0007,\alpha =6.6\times 10^{-5}\;mM\;\cdot \;pA^{-1}\;\cdot \;ms^{-1},\gamma =500\;pA^{2}$$

#### Synaptic inputs and calcium dynamics

Synaptically triggered increases in cytosolic Ca^2+^ directly activate *I*_CAN_. We coupled the synaptic variable *s* to the Ca^2+^ equation. The synaptic variable *s* represents both ionotropic and metabotropic glutamatergic receptor (mGluR) activation. Cytosolic Ca^2+^ changes are attributable to influx through voltage-gated Ca^2+^ channels evoked by AMPA receptor-mediated depolarization as well as group I mGluRs that evoke intracellular Ca^2+^ release from stores in the endoplasmic reticulum (Pace et al., 2007b; Pace and Del Negro, 2008). Rather than explicitly model the biophysics and intracellular signaling that elevate intracellular Ca^2+^, we abstracted the process such that when a presynaptic neuron discharges an action potential, its corresponding synaptic gating variable *s* increments, which then raises intracellular Ca^2+^ in all the postsynaptic neurons to which it projects. The parameter *k*_IP3_ governs how much Ca^2+^ increases per unit increment in *s*. Variable *s* also appears in the chord-conductance equation for *I*_syn_, where it controls AMPA receptor gating.

#### Parameters

The model maintains rhythmic function over substantial variation in $g_{CAN}$, $g_{Na-P}$, $k_{IP3}$, $g_{syn}$, $r_{pump}$, and $k_{Ca}$ from nominal baseline values when $I_{pump}$ is present. Initial values for the membrane potential and gating variables were set constant. The parameters $g_{CAN}$, $g_{leak}$, and overall $g_{syn}$ were randomly assigned from Gaussian distributions given the average and SD listed above. Then, $g_{syn}$ was normalized by the in-degree of each neuron so that the maximum synaptic conductance was equal in all constituent neurons.

### Network simulations

We modeled the preBötC as a directed network because excitatory chemical synapses, rather than gap junctions, are central to its rhythmic function (Greer et al., 1991; Funk et al., 1993; Rekling et al., 2000; Wallén-Mackenzie et al., 2006). Having no empirical information about network topology, we applied an Erdó́s-Rényi random graph (Newman et al., 2001) as the underlying model of preBötC network structure.

Erdó́s-Rényi random networks were generated via two parameters, the network size *n* and connection probability *p*. We denote the underlying graphs as G(*n*,*p*) (Gilbert, 1959). Population size was fixed at *n* = 330 Dbx1 neurons (Hayes et al., 2012; Wang et al., 2014). Excitatory chemical synapses were reported in 13% of paired whole-cell patch-clamp recordings from putatively rhythm-generating preBötC neurons in mouse slices (Rekling et al., 2000), and thus we used *p* = 0.125.

We simulated the network models on the Sciclone computing complex at the College of William & Mary, which features 193 nodes with a total of 943 central processing unit cores, 5.9 terabytes of physical memory, 220 terabytes of disk capacity, and peak performance of 21.2 teraflops. We used a Runge–Kutta fourth-order numerical integration routine with fixed time step of 0.25 ms. Network models were subjected to 100 random deletions because a tally of less than 100 Dbx1 neuron laser ablations was experimentally demonstrated to silence spontaneous respiratory rhythm in an experimental slice model of breathing (Wang et al., 2014). For simulations, one neuron was deleted every 25 s (simulated time). Neuron deletions were achieved by setting the synaptic state variable and its corresponding differential equation to zero, thereby disconnecting the cell from the remaining network. All cumulative neuron deletion simulations were conducted on NeuronetExperimenter, software that simulates large sets of biological neurons arranged with arbitrary network connectivity (http://sourceforge.net/projects/neuronetexp/). All following analyses were conducted on MATLAB (R2015a, MathWorks).

A running time spike histogram provided a convenient measure of ensemble network activity, akin to the experimental recording of respiratory-related hypoglossal nerve (XII) root discharge *in vitro* (Smith et al., 1991; Funk and Greer, 2013). The histogram counted the number of spikes per 10 ms bin, where bins were contiguous for the duration of the simulation. Neurons no longer contributed to the running time spike histogram after ablation.

Transient glutamatergic stimulation of constituent model neurons mimicked a holographic glutamate uncaging protocol applied to preBötC neurons (Kam et al., 2013a). Focal stimulation was achieved by setting the synaptic state variable to 0.6 for 200 ms, without modifying the differential equation, leading to an exponential relaxation of glutamatergic excitation.

### Static network (topological) analysis

The connectivity of a network is reported by the entries in its adjacency matrix *A*, where *A_ij_ =* 1 if and only if there is a directed link (synaptic connection) from neuron *i* to neuron *j*; otherwise, *A_ij_* = 0. In discrete simulations that examine only the topological structure of the underlying graph G(*n*,*p*), without considering the dynamics of nodes and links (i.e., neurons and synapses), ablations were modeled by removing nodes from the network along with their links. We computed three global metrics (*K*-core, number of strongly connected components, as well as the average in/out degree) at the initial state of the network and for its corresponding state after each one of a sequence of 100 random deletions were performed. Also, for each deleted node, we computed four local metrics (local cluster coefficient, closeness centrality, and betweenness centrality) to indicate the importance of the node that was (in each case) removed from the network.

#### Local cluster coefficient

It measures how close the neighbors of the node are to being a complete directed graph, i.e., a graph where each node is connected to every other node. For a node *v_i_* with *k_i_* links, the local cluster coefficient is defined as follows:

$$C_{i}=\frac{\left|\left\{A_{jk}\;:\;v_{j},v_{k}\in N_{i},A_{jk}=1\right\}\right|}{k_{i}(k_{i}-1)},$$

where *N_i_* is the neighborhood of *v_i_*, (the subgraph formed by all the nodes *v_i_* connects to, that is, all the out-neighbors of *v_i_*; Watts and Strogatz, 1998). The numerator is the number of actual connections within *N_i_*, whereas the denominator is the number of connections if *N_i_* is a complete directed graph.

#### Closeness centrality

The farness of node *v_i_* is defined as the sum of shortest paths from *v_i_* to every other node reachable by *v_i_* along directed paths in the network. Closeness of *v_i_* is the reciprocal of the farness and closeness centrality is simply the product of closeness and the number of nodes *n* (Sabidussi, 1966).

#### Betweenness centrality

It measures the frequency that a node acts as a bridge in the shortest path between two other nodes, according to the following formula:

$$C_{B}\left(v\right)=\underset{s\neq v\neq t}{\sum }\frac{\sigma_{st}(v)}{\sigma_{st}},$$

where *s*, *v*, and *t* are three different nodes in the graph G(*n*,*p*), $\sigma_{st}(v)$ is the number of shortest paths between *s* and *t* through *v*, while $\sigma_{st}$ is the total number of shortest paths between *s* and *t* (Newman, 2005)_._ Betweenness centrality is usually normalized by dividing by the number of total possible node pairs (*n*–1)(*n*–2) (excluding *v*).

#### Strongly connected components (SCC)

The strongly connected components of a directed graph G(*n,p*) are subgraphs in which there is a path, in both directions, from each node to every other node (Diestel, 2010). Therefore the number of SCC can exceed unity. Nonetheless, when SCC = 1 the existing network is said to be fully connected, i.e., there are no isolated islands and every node can connect to every other node via a finite number of directed links.

#### *K*-core

It refers to the maximum subgraph whose constituent nodes have at least *k* links (connections), where an incoming synaptic connection and an outgoing synaptic projection are equivalent in terms of counting links.

#### Average in and out degree

Network connectivity of a directed graph G(*n*,*p*) is represented by the entries in its adjacency matrix *A*(*n* × *n*). The sum of the *i*th row $d_{i}^{out}=\overset{n}{\underset{j=1}{\sum }}A_{ij}$ is the out-degree of the *i*th node, whereas the sum of the *i*th column $d_{i}^{in}=\overset{n}{\underset{j=1}{\sum }}A_{ji}$ is the in-degree for the *i*th node. Thus, the average in- and out-degree are given by $\mathrm{(1/n)}\overset{n}{\underset{i=1}{\sum }}d_{i}^{in}$ and $(1/n)\overset{n}{\underset{i=1}{\sum }}d_{i}^{out}$, respectively.

### Active subnetwork analysis

To explore the interaction between network structure and neuronal dynamics, we examine network topology at various time points during simulations by taking a snap shot of the network and analyzing the states of the neurons and synaptic interconnections (directed links). If a structural connection from neuron *i* to *j* (i.e., *A_ij_* = 1) exists but the excitability of presynaptic neuron is below some activity threshold, then ostensibly the connection is nullified because it has no postsynaptic impact. We define the subnetwork that considers only active synapses to be the functionally active subnetwork, thereby modifying the actual network structure according to physiological dynamics.

Synaptic excitation evokes the inward current *I*_CAN_, which influences burst generation in the Rubin–Hayes model. The model also includes inward currents *I*_Na-P_ and *I*_syn_. We examined active subnetworks based on *I*_CAN_, *I*_Na-P_, *I*_syn_, and *s* (which gates *I*_syn_, and indirectly, *I*_CAN_).

Each simulation consists of *N* bursts before rhythm cessation, with peak times (*t_x_*) of the inspiratory-like bursts (*t_1_, t_2_, …, t_N_*) and the periods (*T_x_*) between them (*T_1_, T_2_, …, T_N-1_*). We define an analytic window equal to $\min(T_{x-1},T_{x})$, where x∈[2,N−1], centered at *t_x_*. The last time window is equal to *T_N-1_*, centered at *t_N_*. For each neuron at each time step, we compute the average *I*_CAN_ (in units of pA) over its corresponding analytic window. Although peak *I*_CAN_ transiently reaches 9–15 nA, the average *I*_CAN_ over the duration of the analytic window is much smaller, ranging from 0 to 10 pA. Then, we identify subsets of neurons whose *I*_CAN_ exceeds a series of arbitrary equidistant thresholds (−2 pA, −2.25 pA, …, −5.5 pA) within that time window, which we define as the active subnetwork. At any given level set for *I*_CAN_ thresholds, we sorted neurons according to their appearance (or absence) in the active subnetwork. By varying the thresholds systematically, we rank-ordered the neurons according to the frequency of their appearance in the active subnetwork. Then, we selectively ablated neurons in the sequence of their active subnetwork rank order.

## Results

We simulated the preBötC using networks of Rubin–Hayes model neurons whose underlying connectivity was described by Erdó́s-Rényi random graphs G(*n,p*) with network size *n* = 330 and synaptic connection probability *p* = 0.125. We determined these values after searching (*n*,*p*) parameter space for rhythmic networks whose behavior matched the respiratory rhythm in slice preparations (Rekling et al., 2000; Hayes et al., 2012; Wang et al., 2014). *E*_L_ and *g*_Na-P_ were fixed at −61.46 mV and 1 nS, respectively, because these parameters are physiologically realistic and within the operating range of the original Rubin–Hayes model (Rubin et al., 2009), as well as the modified version that includes *I*_Na-P_ (Dunmyre et al., 2011). At the start of each simulation (given *n*, *p*, *E*_L_, and *g*_Na-P_ as described above) the networks were spontaneously rhythmic but sequentially deleting constituent neurons slowed and then irreversibly stopped the rhythm. The cumulative tally to stop the rhythm was 39.1 ± 13.2 (mean ± SD), which represents 11.8% of the network (*n* = 15 simulations).

**Figure 1.**
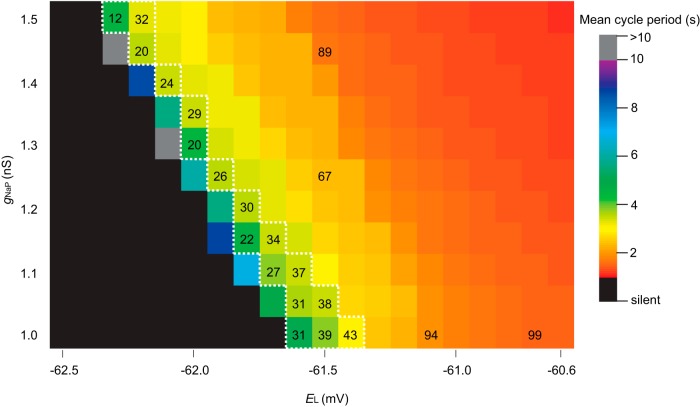
Networks of Dbx1 preBötC neurons with various *E*_L_ and *g*_Na-P_. Blocks show the mean cycle period according to the colorimetric scale (right) for one 30 s simulation on the same network realization without any neuron deletions of each (*E*_L_, *g*_Na-P_) pair. Ablation tallies on representative parameter sets are indicated on corresponding blocks. The dotted white line encloses all (*E*_L_, *g*_Na-P_) pairs that produce a mean cycle period of 3.5–5 s.

The above tally underestimates the experimental tally by approximately one-half, 85 ± 20. Although we proposed that premotor neurons in the preBötC, which the model lacks, could explain, at least in part, this model-experiment disparity (Wang et al., 2014), we did not previously investigate whether the excitability parameter *E*_L_ or the conductance *g*_Na-P_ influenced the ablation tally. To do so here, we first tested how *E*_L_ and *g*_Na-P_ influence network behavior. Using the same network realization, i.e., the same underlying G(*n,p*) structure, we simulated networks with *E*_L_ spanning from −60.6 to −62.5 mV (*E*_L_ outside this range is not physiologically realistic) and *g*_Na-P_ spanning from 1 to 1.5 nS (*g*_Na-P_ < 1 nS is not physiologically realistic). Simulations ran for 30 s absent neuron deletions to quantify network rhythmicity (Fig. 1; blocks are color-coded for cycle period). Lowering either *E*_L_ or *g*_Na-P_ slowed down the rhythm, such that for some (*E*_L_, *g*_Na-P_) pairs the rhythm stopped (Fig. 1, black squares) and for some the period would exceed 10 s (Fig. 1, grey squares), whereas elevating *E*_L_ or *g*_Na-P_ had the opposite effect (it speeds up the rhythm) such that for some (*E*_L_, *g*_Na-P_) pairs the cycle period was ∼1 s (Fig. 1, red squares). Networks along the diagonal (Fig. 1, within the dotted white line) reflect the set of (*E*_L_, *g*_Na-P_) pairs whose networks produce experimentally reasonable cycle periods of 3.5–5 s. The ablation tally did not vary systematically along this diagonal (29.1 ± 8.0, mean ± SD, *n* = 17) when the network was subjected to the same neuron deletion sequence. However, cumulative ablation experiments performed on networks to the right of this diagonal resulted in much faster cycle periods (∼1 s) and notably higher ablation tallies (Fig. 1, orange and red squares with ablation tallies). These results indicate that the ablation tally depends on the initial cycle period such that the initial period could be treated as a proxy for the network robustness. Furthermore, these results indicate that (*E*_L_, *g*_Na-P_) combinations that yield networks with cycle period in the range 3.5–5 s are equally sensitive to cumulative cellular ablation.

Given that various (*E*_L_, *g*_Na-P_) pairs produce realistic network rhythms, and the trade-off between *E*_L_ and *g*_Na-P_ results in commensurate ablation tallies, we used *g*_Na-P_ = 1 nS, which is consistent with the richest set of dynamical behaviors (including voltage-dependent bursting-pacemaker activity) in the Rubin–Hayes model as extended by Dunmyre et al. (2011) and *E*_L_ = −61.46 mV, which straddles the two blocks on the diagonal line in Figure 1.

### Cell ablation stops rhythm with no precipitous drop in network connectivity

**Figure 2.**
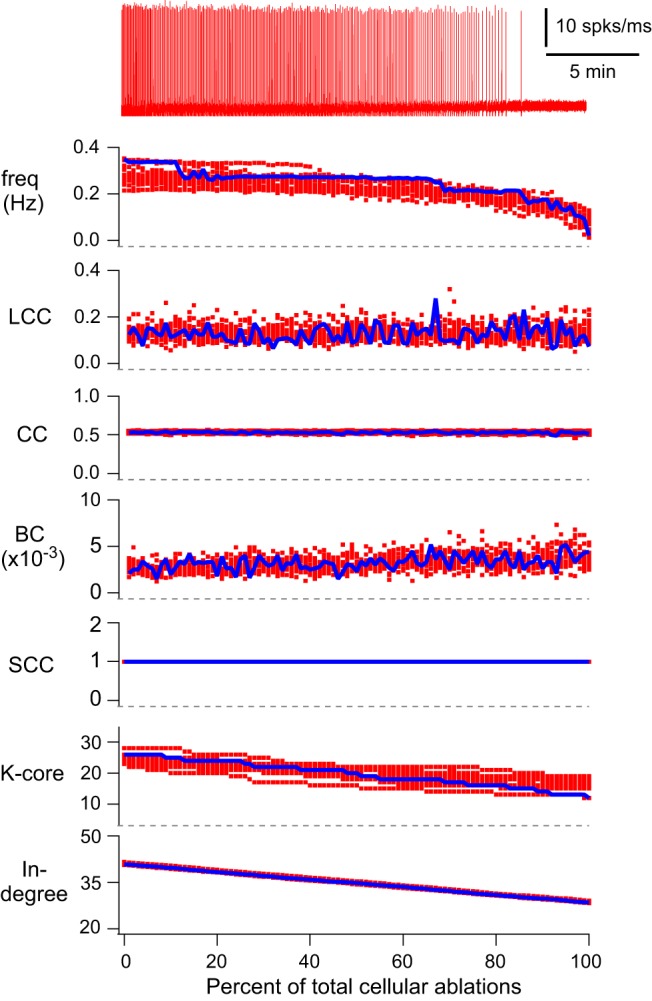
Cumulative cellular ablations in the model preBötC network. Running time spike histogram (top, red) and plots of rhythmic burst frequency and six discrete network metrics (global and local). The simulated experiment where a total of 100 neurons were deleted (one per 25 simulated seconds) in sequence was repeated 15 times. The running time spike histogram is shown for one representative simulation. The top trace shows inspiratory-like burst frequency (Hz) for all 15 simulations. The abscissa (percentage of total cellular ablations) is the same for frequency and all discrete network metrics. LCC, CC, and BC are plotted for each neuron in the deletion sequence. The number of SCCs, *K*-core and in-degree are plotted for neurons in the remaining network during the ablation sequence. Blue symbols show the average metric (for 15 simulations) during the deletion sequence; these quantities were no longer computed after the 100th ablation. Red symbols show the scattered data points for all individual 15 simulations.

Previously, we reported canonical local and global measures of topology for the underlying graph G(*n*,*p*) at the start of a simulation and after piecewise cellular deletions stopped the rhythm (Wang et al., 2014, their supplemental file 2). Here, we provide more detail by tracking the state of network topology as a function of cumulative percentage of total ablations (Fig. 2). During progressive ablation sequences, there were no major changes in measures of local connectivity, i.e., local metrics, such as local cluster coefficient (LCC), closeness centrality (CC), and betweenness centrality (BC). A network is strongly connected if a directed path exists between any two constituent nodes, which can be quantified by the number of strongly connected components (SCC). Cumulative deletion sequences at no point caused SCC to depart from unity (a fully connected graph). Other global connectivity metrics, such as the *K*-core and the average in-degree, showed linear declines. Whereas network burst frequency declined to zero in each simulation, *K*-core remained >12 and the average in-degree remained >28. These data indicate that the model networks remain fully connected for the entire duration of cumulative cellular ablation simulations that invariably stop rhythmic function.

The linear drop in average in-degree and *K*-core are logical effects due to cumulative neuron deletions from the network. However, we are unable to fully identify their dynamics effects unless we consider the loss of network structure in tandem with the neuronal dynamics.

### Active subnetwork analysis

**Figure 3.**
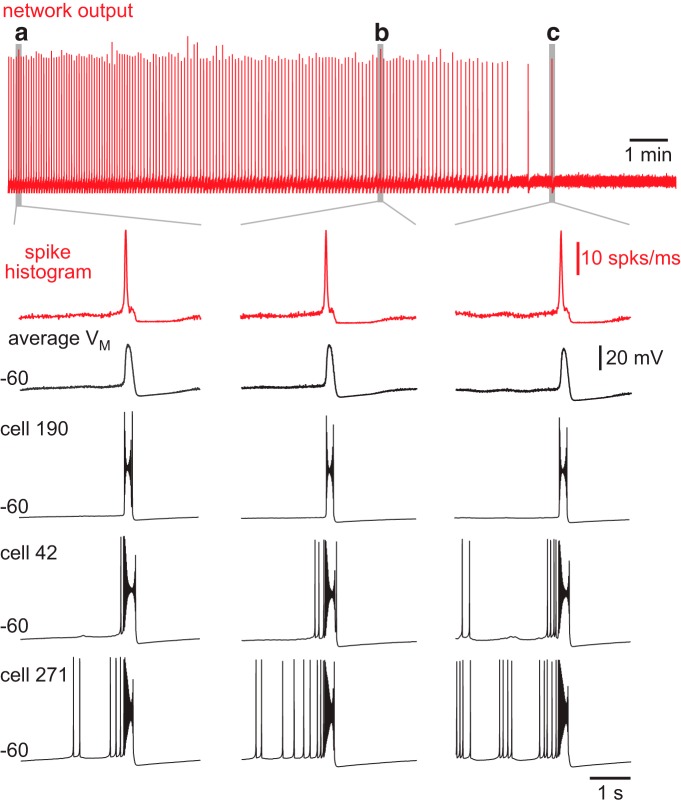
Model network bursts do not diminish during ablations sequences. Running time spike histogram (top, red) for one experiment. Time calibration (1 min) is displayed. ***a***, The fifth network-wide burst. ***b***, A network-wide burst after 21 deletions. ***c***, The last network-wide burst after 31 deletions. The running time spike histogram (vertical calibration 10 spikes/ms) and average membrane potential (*V*_M_, vertical calibration 20 mV) for all remaining neurons in the network for network-wide bursts (***a***, ***b***, and ***c***) are shown at higher sweep speed. Cells 190, 42, and 271 from the model system are shown individually. Baseline membrane potential (–60 mV) and time calibration (1 s) apply to all traces.

Next we addressed how cumulative ablations affect network excitability, which encompasses a range of cellular and synaptic factors. Network burst output, which was quantified by the running time spike histogram (Fig. 3, red traces), did not decline during the ablation sequence. Figure 3 shows three individual bursts, including the final one, with faster sweep speed to emphasize their similarity. Likewise, there was no systematic drop in baseline membrane potential, as quantified by the average voltage trace for all constituent neurons in the remaining network, and shown for three representative cells (Fig. 3, black traces).

Because the topology of the underlying graph G(*n*,*p*) remained strongly connected (Fig. 2) and network burst output did not decline (Fig. 3), we sought a more fine-grained analysis to identify how cumulative cellular ablations changed the system such that it stopped its autonomous rhythmic function. To begin we exploited an obvious property of the nervous system: neurons may share a synaptic connection, but unless the presynaptic partner is spiking and EPSPs are registered in the postsynaptic partner, then their connection is not active. Conversely, an active synaptic connection features a presynaptic neuron whose spikes trigger EPSPs in the postsynaptic partner.

Thus we analyzed active subnetworks consisting of synaptically engaged partners as defined above. For this analysis, spike generation indicated presynaptic activity, with corresponding postsynaptic activity gauged by four different metrics related to *I*_CAN_, *I*_Na-P_, *I*_syn_, and state variable *s*.

**Figure 4.**
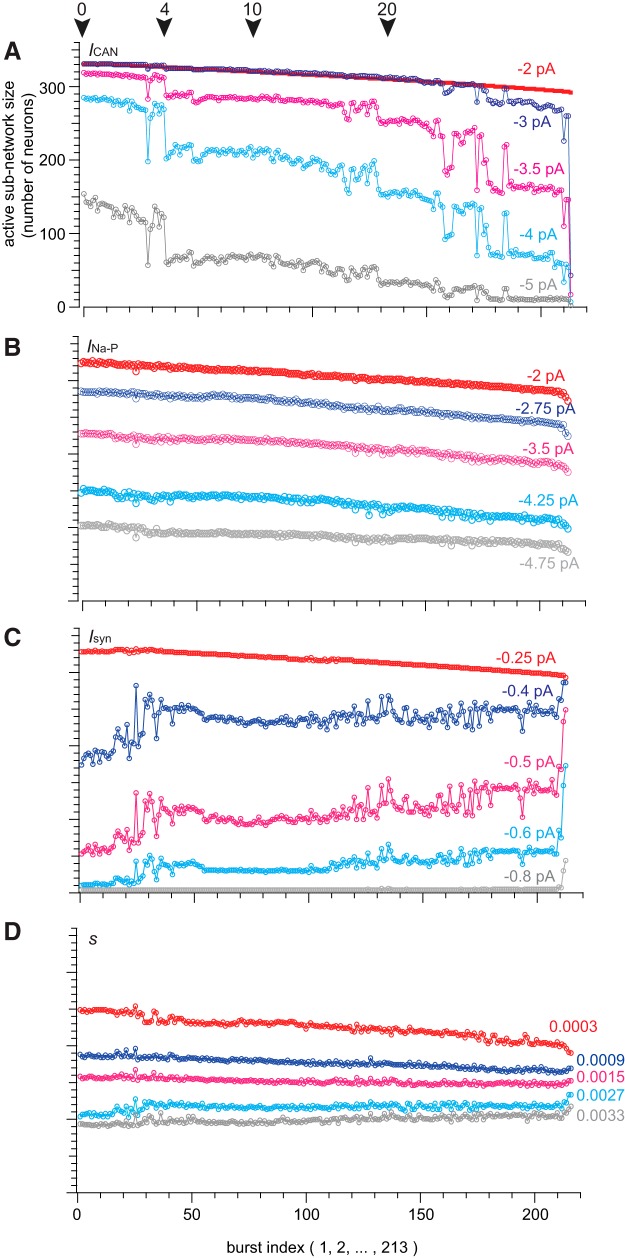
Active subnetwork properties for a sequence of inspiratory-like bursts given different *I*_CAN_ thresholds. ***A***, Active subnetwork size (number of neurons) for five representative *I*_CAN_ thresholds (indicated above each trace) plotted versus sequential burst indices (1–213) for one simulation. Color represents different threshold values. Arrows indicate deletions 0, 4, 10, and 20. ***B***, Active subnetwork size (number of neurons) for five representative *I*_Na-P_ thresholds (indicated above each trace) plotted versus sequential burst indices (1–213) for one simulation. ***C***, Active subnetwork size (number of neurons) for five representative *I*_syn_ thresholds (indicated above each trace) plotted versus sequential burst indices (1–213) for one simulation. ***D***, Active subnetwork size (number of neurons) for five representative *s* thresholds (indicated above each trace) plotted versus sequential burst indices (1–213) for one simulation.

**Figure 5.**
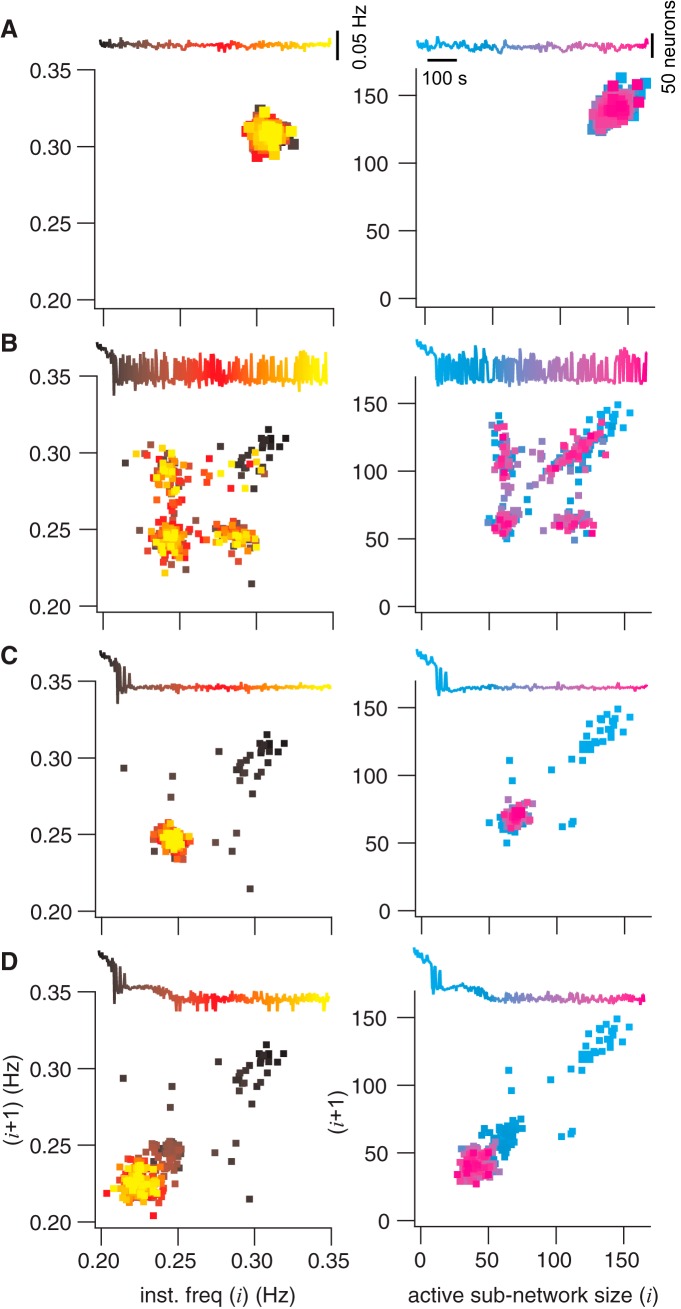
Poincaré maps of instantaneous burst frequency and active subnetwork size. Each panel shows the map for frequency or active subnetwork size for the cycle *i* + 1 plotted versus the prior cycle (*i*). Frequency maps are at left (black to yellow). Active subnetwork size maps are at right (cyan to magenta). Each Poincaré map features an inset of the time series, where temporal relations are color-coded to points in the map. Vertical calibrations are given in ***A***. ***A***, Poincaré maps without any neuron deletions. ***B***, ***C***, and ***D*** show the same information for the same network realization as ***A***, where the ablation tally was frozen after 4, 10, or 20 ablations, respectively.

*I*_CAN_ was formulated as a synaptically triggered inward current based on experimental evidence (Crowder et al., 2007; Pace et al., 2007b; Mironov, 2008, 2013; Pace and Del Negro, 2008; Rubin et al., 2009; Mironov and Skorova, 2011). Its activation depends proximally on cytosolic Ca^2+^, which rises because of synaptic drive from presynaptic partners. Once activated, *I*_CAN_ generates postsynaptic bursts. Therefore *I*_CAN_ is a cellular parameter whose magnitude depends both on the number of presynaptic partners and their activity (topology and dynamics). Taking the average value of *I*_CAN_ for each neuron over an analytic time window centered at the peak of each inspiratory burst (see Materials and Methods for full definition), we generated a time series of active subnetwork snapshots spanning the simulation. All the constituent neurons whose average *I*_CAN_ exceeded some threshold value comprised the active subnetwork. Figure 4*A* plots the size of the *I*_CAN_ active subnetwork for five different thresholds (−2, −3, −3.5, −4, and −5 pA) during the course of one representative simulation. Note, *I*_CAN_ may transiently exceed 9 nA, but its average over the entire analytic window is much lower, thus threshold is 1000-fold less than peak *I*_CAN_. Cumulative cell ablation caused the *I*_CAN_ active subnetwork size to fluctuate and progressively diminish until the rhythm stopped. *I*_CAN_ active subnetwork size often locked onto a particular value, and remained there despite ongoing cellular ablations, then fluctuated between levels, before finally locking onto a new smaller size. Although not illustrated in Figure 4, each decrement of the *I*_CAN_ active subnetwork size was accompanied by a corresponding decrease in burst frequency (further explained below and in Fig. 5).

We further analyzed active subnetworks based on *I*_Na-P_, *I*_syn_, and *s* using the same network realization and cell-deletion sequence. A range of threshold values for *I*_Na-P_, *I*_syn_, and *s* were used to compute active subnetwork size (Fig. 4*B*–*D*).

*I_Na-P_* transiently reaches several hundred picoamperes but its average over the analysis window is much lower. We used a threshold range from ∼0 to −8.0 pA (−2, −2.75, −3.5, −4.25, and −4.75 pA are shown in Fig. 4*B*). Regardless of threshold, the *I_Na-P_* active subnetwork size declined linearly without precipitous changes during the ablation sequence. These results suggest that *I_Na-P_* is not diagnostic for a breakdown in network function.

*I_syn_* also transiently measures >100 pA but its average over the analysis window is much lower. We used a threshold range from −0.25 to −1.2 pA (Fig. 4*C* shows −0.25, −0.4, −0.5, −0.6, and −0.8 pA because larger thresholds produced active subnetworks of size zero). *I*_syn_ active subnetwork size showed stepwise increases for intermediate thresholds with more noise compared with the *I*_CAN_ active subnetwork. These increases occurred at approximately the same time points where the *I*_CAN_ active subnetwork decreased in size, which suggest that deficits in *I*_CAN_ (and the *I*_CAN_ active subnetwork) caused by cell ablation are partially compensated by ionotropic synaptic current *I*_syn_ (and the *I*_syn_ active subnetwork).

In the case of the synaptic gating variable *s* (all thresholds on the unitless interval [0,1]), no network degradation was seen for any threshold. Only a slight increase occurred when the network-wide rhythm approached the rhythm termination. These results suggest that *s* is not diagnostic for a breakdown in network function.

**Figure 6.**
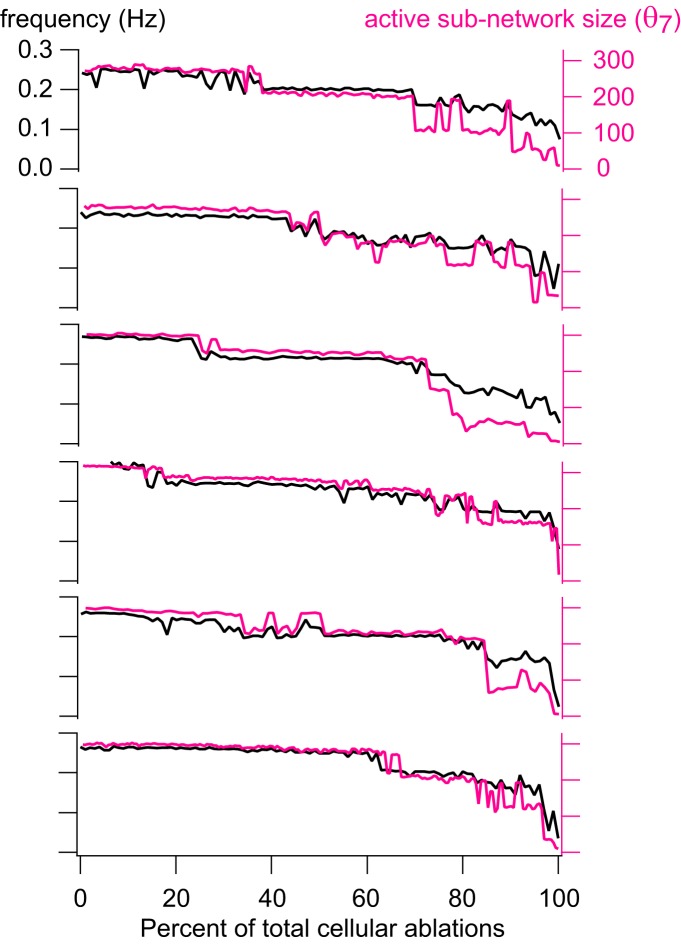
Group data for instantaneous frequency and active subnetwork size. Instantaneous frequency (Hz) and active subnetwork size (number of neurons) at threshold θ_7_ = –3.5 pA are plotted for cumulative neuron deletion simulations on six different network realizations. Black traces show instantaneous frequency (Hz); red traces show active subnetwork size (number of neurons) for each cycle period, plotted versus the percentage of total cellular ablations (%).

It may seem counterintuitive that the network burst output did not change during the ablation sequence (Fig. 3, red traces), whereas the *I*_CAN_ active subnetwork size decreased stepwise (Figs. 4*A*, 5, as well as *n* = 6 simulations shown in Fig. 6). Although the average *I*_CAN_ declined during the sequence, network burst output remained stable because *I*_CAN_ has a biphasic influence on the ability to generate action potentials; intra-burst spiking decreases when *I*_CAN_ is too low or too high. *I*_CAN_ generally ensures that inspiratory bursts remain more robust and larger in magnitude than is needed to sustain rhythmogenesis (Kam et al., 2013b). However, the ability of *I*_CAN_ to enhance burst magnitude causes depolarization block of spiking when its magnitude is large, which cuts down on the number of spikes per burst (Rubin et al., 2009; Fig. 3, example cells). Thus, as the average *I*_CAN_ decreases during the cumulative cell ablation sequence, neurons with low *g*_CAN_ decrease burst amplitude and generate fewer intra-burst spikes, whereas other neurons with larger *g*_CAN_ generate more intra-burst spikes because the ability of *I*_CAN_ to cause depolarization block of spiking is reduced during the course of the ablation sequence. As a result, the network burst output, as quantified by the running time spike histogram, does not decline.

To further investigate the properties of the *I*_CAN_ active subnetwork during constituent neuron deletions, we repeatedly simulated the exact same network realization (same as Fig. 4, starting from the same initial conditions) but we stopped the deletion sequence after 0, 4, 10, and 20 ablations and then continued the simulation for 1025 s to observe steady-state behavior. Figure 5 shows Poincaré maps of instantaneous burst frequency (left column) and *I*_CAN_ active subnetwork size (right column) with a corresponding time series of network activity (insets). Points in the Poincaré maps are color-coded according to elapsed time in the series.

Under the circumstance of no neuron deletions (Fig. 5*A*), instantaneous frequency and *I*_CAN_ active subnetwork size remain tightly clustered, which shows the dynamics of the network fluctuating around a limit cycle. A representative sample shows a point at 0.308 Hz burst frequency, where the active subnetwork size measures 140.

That steady state is impaired after only four deletions. The *I*_CAN_ active subnetwork then alternates between the former steady state and a new lower state with representative instantaneous frequency of 0.247 Hz and active subnetwork size of 67 (Fig. 5*B*). The Poincaré map shows that these two states are repeatedly visited throughout the simulation (note the spread of color coding in the Poincaré map for the corresponding time series).

When six more neurons are deleted (for a total of 10), the network completes its transition to the (0.247 Hz, 67) low state (Fig. 5*C*), and the Poincaré map homes in on the lower state that first appeared in the four-deletion case (note the yellow and magenta points are concentrated at the low state).

This steady state (0.247 Hz, 67) remains the sole periodic attractor for the system despite subsequent cellular ablations 11–19. Nevertheless, another transition occurs after a total of 20 neurons are deleted, leading to a new steady state with representative instantaneous frequency of 0.223 Hz and an active subnetwork of 39 neurons (Fig. 5*D*).

These analyses demonstrate that cumulative deletions diminish the *I*_CAN_ active subnetwork size in tandem with instantaneous frequency via state-flickering and step-like transitions. This dynamical behavior observed when passing through critical thresholds is seen in many other systems, from ecosystems to financial markets (Scheffer et al., 2009). Figure 6 shows six different network realizations to illustrate that step-like decreases in the active subnetwork size and frequency characterize how the system generally behaves in response to piecewise disassembly, i.e., cumulative cellular ablation.

### Role of *I*_CAN_ in model network bursts

**Figure 7.**
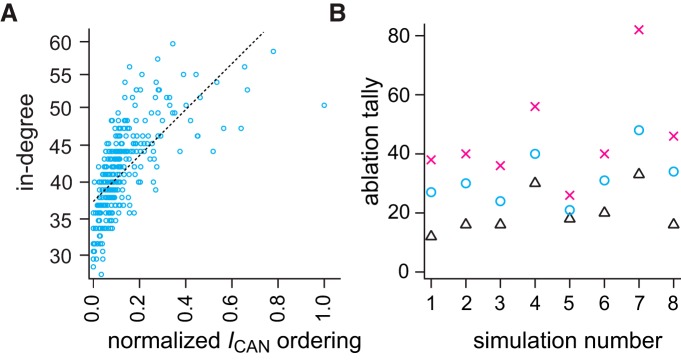
In-degree correlates with normalized *I*_CAN_ ordering and targeted ablation tallies. ***A***, Linear regression between in-degree (unitless) and normalized *I*_CAN_ ordering among neurons in the same network. *I*_CAN_ order was computed based on the maximum number of appearances in the active subnetwork given 15 different thresholds. Blue symbols show the scattered distribution of in-degrees and normalized *I*_CAN_ ordering. Linear fit is shown by a dotted line. ***B***, Ablation tallies (number of neurons) for three deletion strategies on different network realizations (*n* = 8). X-symbols mark the tally from eight different simulations where low *I*_CAN_-order neurons were selectively ablated. Triangles mark when high *I*_CAN_-order neurons were selectively ablated. Circles mark the tally for random neuron deletions (control default strategy).

*I*_CAN_ generates inspiratory bursts in the Rubin–Hayes model. Therefore, it is straightforward to predict that neurons with greater *I*_CAN_, which appear more frequently in the active subnetwork, play a more important role in rhythmogenesis. We ordered the neurons according to average *I*_CAN_, which was correlated with in-degree, the number of presynaptic partners (Fig. 7*A*). Large in-degree did not represent a greater overall synaptic conductance because *g*_syn_ was scaled according to total number of inputs, i.e., the product of in-degree and *g*_syn_ was uniform among neurons (see Materials and Methods). To test whether neurons with larger *I*_CAN_ were more important for rhythmogenesis, we ablated neurons according to *I*_CAN_ ordering (instead of randomly). Figure 7*B* shows eight cumulative-ablation simulations (8 different network realizations, eight random deletion sequences, *n* = 8) in which targeting neurons high in the *I*_CAN_ ordering systematically decreased the ablation tally (black triangles, 20±7) required to stop the rhythm compared to random deletions (cyan circles, 32±9). Conversely, targeting neurons low in the *I*_CAN_ ordering systematically raised the ablation tally (magenta X’s, 46±17) to stop the rhythm. A standard one-way ANOVA showed that there was a statistically significant effect (α = 0.05) of targeting condition on mean ablation tally (*F* = 9.17, *p* = 0.0014). *Post hoc* comparisons using the Tukey HSD test indicated that the mean ablation tally for the higher *I*_CAN_ ordering was significantly different than the lower *I*_CAN_ ordering (mean difference = 25.38, SD = 14.95, *p* = 0.0009). However, the ablation tallies of random deletion sequences did not significantly differ from the higher *I*_CAN_ ordering (mean difference = 11.75, SD = 14.95, *p* = 0.14). We interpret these data to indicate that the neurons with higher *I*_CAN_ tend to play a more important role, and that deleting such neurons damages the overall ability to spontaneously generate network bursts. Conversely, neurons with lower *I*_CAN_ play a less crucial rhythmogenic role, and their selective ablation causes less deleterious network effects.

*I*_CAN_ active subnetwork analyses of targeted cumulative ablation simulations (Fig. 7*B*, black triangles) were qualitatively identical to the active subnetwork analyses for random deletion simulations except for their lower tally (data not shown). Furthermore, the global and local metrics for the underlying graph G(*n*,*p*) were also the same in targeted cumulative ablation simulations and were indistinguishable from the results in Figure 2 when plotted along the same abscissa (percentage of total cellular ablations) which normalizes for the lower ablation tally in targeted cumulative ablation simulations.

### Recurrent excitation and pre-inspiratory latency

To investigate recurrent excitation, we tracked each neuron from its quiescent post-burst baseline membrane potential until the peak of the subsequent inspiratory burst. Many neurons begin spiking prior to the inspiratory burst (Fig. 3). Early activation during the pre-inspiratory phase (i.e., pre-inspiratory latency) has been hypothesized to be a key rhythmogenic property for 25 years (Smith et al., 1990; Rekling et al., 1996; Rekling and Feldman, 1998). Pre-inspiratory latency depends overwhelmingly on input resistance; preBötC neurons with low *g*_leak_ tend to spike early in their pre-inspiratory phases (Del Negro et al., 2002a; Koizumi and Smith, 2008).

**Figure 8.**
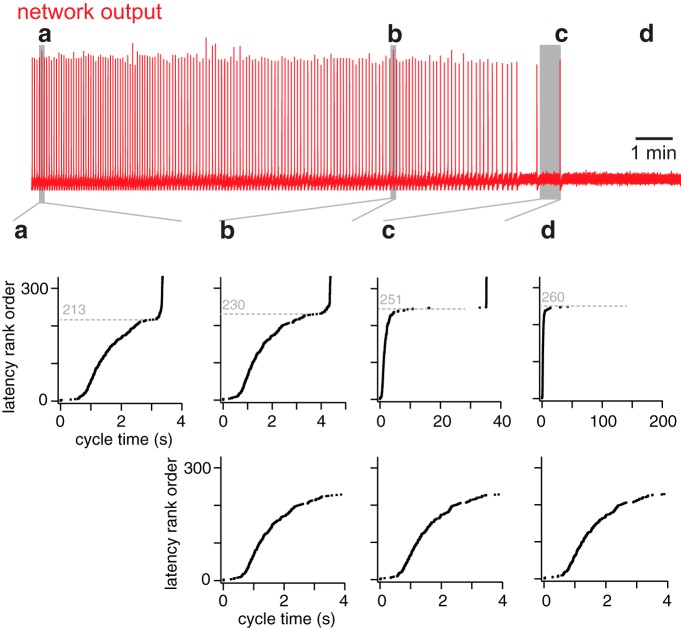
Latency rank order of all constituent neurons in the network at four specific time points in a simulation. Top (red), The running time spike histogram for a random neuron deletion simulation. ***a***, ***b***, and ***c*** indicate three cycles leading to network-wide bursts (at time points 16, 557, and 814 s, respectively). ***d***, The time after the last burst. The middle shows the latency rank order (defined in Results) for cycles ***a***, ***b***, and ***c***, and after rhythm termination (***d***) plotted versus cycle time (in seconds). The dotted line indicates latency rank order (unitless) of the neuron after which the curve inflects upward, leading to a network-wide burst. The lower panel shows the same data as the middle, but where cycle time is limited to 0–4 s, emphasizing the similarity of ***b***, ***c***, and ***d***.

Figure 8 shows constituent neurons sorted by pre-inspiratory latency for the same network realization as in Figures 3–5. Latency rank order (earliest activating neurons obtain lower rank) is plotted versus activation cycle time, i.e., when the first action potential occurs during the inter-burst interval. Latency for constituent neurons 1 to ∼210 remains relatively fixed (Fig. 8*a*,*b*–*d*, bottom). Individual network cycles are not identical (Carroll and Ramirez, 2013; Carroll et al., 2013; Kam et al., 2013b) but pre-inspiratory latency for a neuron generally remains within a few tens of milliseconds from cycle to cycle. An early-spiking neuron does not convert to a late-spiking one, and vice versa.

Interneurons low in the latency rank order (i.e., rank order 1–210) spike spontaneously and respond to synaptic input such that by cycle time ∼4 s they are ostensibly all active (Fig. 8*a*,*b*–*d*, bottom). However, before the network-wide burst occurs, all the remaining neurons (i.e., rank order >210) need to be recruited. The recruitment curve inflects upward as excitation spreads to the neurons with highest latency rank order. The network-wide burst occurs where the recruitment curve is vertical. Cumulative neuron deletions shift the inflection point of the recruitment curve to higher and higher latency rank order. Sample bursts at three different time points have their inflection points at neurons with latency rank order 213, 230, and 251, respectively (Fig. 8*a*–*c*, top. Note, that the figure legend reports the time points and ablation tallies that correspond to *a*–*d*). In the process, the time required for the network-wide burst to occur lengthens. This recruitment process can last ∼30 s or more for an extensively lesioned network (Fig. 8*c*, top).

Cumulative ablations ultimately preclude this final transition to the network-wide burst phase. For the 200 s of network activity shown in Figure 8*d* interneurons whose latency rank order exceeds 251 never activate.

### Rate of recurrent excitation

**Figure 9.**
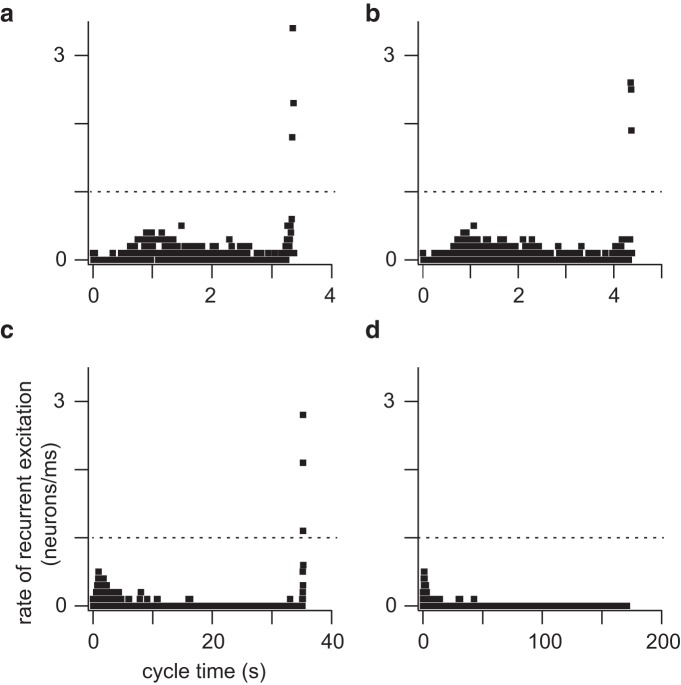
Rate of recurrent excitation (neurons/ms) plotted at four specific time points in a simulation (same time points as Fig. 8). Dotted line in each panel indicates the threshold rate (1 neuron/ms); see text for details.

Cumulative neuron ablation impairs recurrent excitation. To measure this deficit we defined the rate of recurrent excitation during the inter-burst interval to be the number of neurons that emerge from quiescence and spike per millisecond. Note that a constituent neuron may spike during the inter-burst interval but then fall quiescent again, and in that case would not be double counted in our analysis (only the first spike matters). This definition enabled us to measure the speed of propagation of activity throughout the network. We computed the rate of recurrent excitation while the network was still functional, as well as after rhythm cessation by applying a transient stimulus. Whenever the rate of recurrent excitation exceeded 1 neuron/ms a network-wide burst occurred, even when the inter-burst interval was long. However, if the rate of recurrent excitation did not reach 1 neuron/ms, then no network-wide burst could be spontaneously generated (Fig. 9*a*–*d*, which correspond to 8*a*–*d*).

An experimental study showed that glutamate un-caging onto four to nine preBötC neurons evokes inspiratory bursts when the network is quiescent (Kam et al., 2013a), which we later replicated in network models (Wang et al., 2014). To test the hypothesis that a rate of recurrent excitation exceeding 1 neuron/ms evokes network-wide bursts, we computed the pre-inspiratory latency and rate of recurrent excitation for a simulated uncaging experiment, using the network realization from Figure 8.

**Figure 10.**
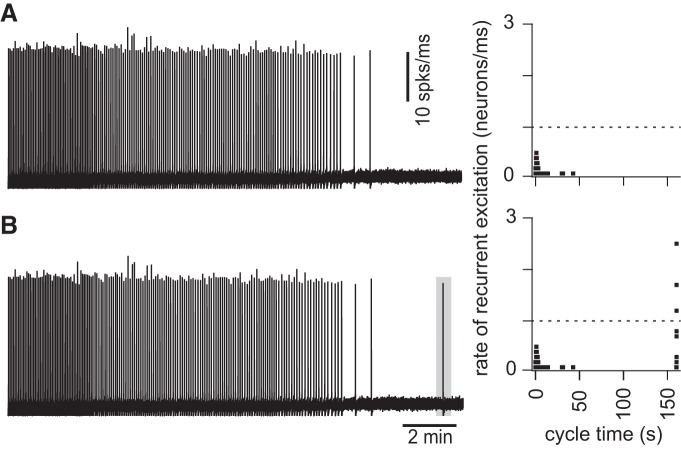
Stimulation of four individual constituent neurons (which remain the network after cumulative ablation stops rhythmicity) evokes a network-wide burst and accelerates the rate of recurrent excitation. For the same network realization and random neuron deletion sequence as in Figures 8 and 9*A*,*B*, running time spike histogram (10 spks/ms) versus cycle time. Time calibration applies to both traces. At right, the rate of recurrent excitation (right) is plotted versus cycle time (s), where the cycle time is reinitialized to zero immediately following the final network-wide burst. The dotted line indicates the threshold rate of 1 neuron/ms (Fig. 9). ***B*** differs from ***A*** only in that four neurons are transiently stimulated 3 simulated minutes after rhythm cessation (see Materials and Methods for details of stimulation).

The rhythm terminated after 32 ablations in that particular network realization. At 975 s the ablated network was no longer spontaneously active (Fig. 10*A*), yet simultaneously and transiently stimulating four neurons (the synaptic gating variable was raised to *s* = 0.6 for 200 ms) evoked a network-wide burst (Fig. 10*B*), showing that the network was capable of generating an inspiratory burst, but not autonomously and without sustainable rhythmicity.

## DISCUSSION

To understand the composition of the core inspiratory central pattern generator, studies performed *in vivo* selectively targeted neurokinin-1 receptor-expressing rhythmogenic preBötC neurons for saporin poisoning, which resulted in a progressive cumulative lesion (Gray et al., 2001; McKay et al., 2005). The animals developed pathological and in some cases fatal breathing phenotypes over the course of several days, during which time saporin killed off a significant fraction of the rhythmogenic preBötC core. It is not possible to know how much of the preBötC was destroyed because neither the network size at the start of those studies, nor the total number of cells killed, was known.

Subsequently, we developed a cell-specific detection and laser ablation methodology to interrogate preBötC network structure and function, as well as establish quantitative cellular parameters that govern its operation (Hayes et al., 2012; Wang et al., 2013, 2014). We reported that cumulative destruction of 85 ± 20 (mean ± SD) interneurons derived from Dbx1-expressing precursors, corresponding to ∼15% of the preBötC core population, slowed and then irreversibly stopped the respiratory rhythm. However, the experiments could not explain why it slowed down and stopped, so we sought an explanation via modeling. The model is experimentally well founded. The Rubin–Hayes model (Rubin et al., 2009) as formulated subsequently by Dunmyre et al. (2011) serves as our preBötC interneuron. Network size (*n*) and connection probability (*p*) were determined empirically (Hayes et al., 2012; Wang et al., 2014). Parameters *g*_Na-P_ and *E*_L_ were selected from a physiologically realistic range of values that yielded networks whose cycle period matched typical slice rhythms; varying these parameters to maintain the cycle period had no undue influence on ablation tally (Fig. 1). The Erdó́s-Rényi configuration was chosen as a starting point. No existing data suggest that preBötC network structure conforms to either scale-free or small-world configurations, which are reasonable alternative paradigms (Watts and Strogatz, 1998; Barabási and Albert, 1999; Newman, 2010).

The model preBötC remains topologically strongly connected in response to cumulative ablation of its constituent neurons, as we determined by a suite of global and local metrics. To more deeply assess the effects of cumulative ablation we had to screen the network for measures of activity: if a presynaptic neuron is spiking and the postsynaptic neuron registers synaptic potentials (which can be monitored via *I*_CAN_, *I*_Na-P_, *I*_syn_, or *s*) then these partners are ostensibly part of the active subnetwork.

These criteria enabled us to quantify how cumulative ablations degrade the size and rhythmic frequency of the system. Active subnetworks based on *I*_Na-P_, *I*_syn_, or *s* did not reveal degradation of the core oscillator network during ablation sequences. The *I*_CAN_ active subnetwork, however, did degenerate progressively. Curiously, the *I*_CAN_ active subnetwork did not degrade smoothly but rather decreased in stepwise transitions. These step-like changes in frequency and size were novel and completely nonintuitive results in a respiratory network modeling study. Although each network realization is unique, step-like degradation of the *I*_CAN_ active subnetwork was a characteristic pattern that occurred in every simulated experiment. The steps occurred at unpredictable intervals such that stable periodic regimes could be maintained during several consecutive cellular ablations before another transition. These transitions often (but not always) exhibited bistability, where the *I*_CAN_ active subnetwork alternated between two states, each having a characteristic active subnetwork size and network-wide burst frequency. These results indicate that the core oscillator defends stable periodic regimes, and can do so over the course of sequential deletions (e.g., ablations 11–19; Fig. 4*A*). Nevertheless, as constituent cells are lost to ablation, the *I*_CAN_ active subnetwork gets progressively smaller and the rhythm slows down in tandem until spontaneous functionality is unsustainable.

Based on previously published modeling results (Wang et al., 2014), this study provides a more complete explanation for rhythm cessation in the preBötC in response to cumulative neuron deletions. Additionally, we define and then analyze active subnetworks to discover that *I*_CAN_ coupled with in-degree is an important factor to explain burst initiation and the sustainability of spontaneous rhythms. Finally, we discovered that the rate of recurrent excitation is the key factor for maintaining spontaneous rhythmogenic function in the network model. By rank ordering the neurons by pre-inspiratory latency, we found that cumulative neuron deletions would decelerate the process of recurrent excitation such that when the rate of recurrent excitation fails to achieve a threshold of 1 neuron/ms, then spontaneous rhythm and burst generation is precluded (further discussed below).

### Cellular factors that contribute importantly to rhythmogenesis

As a synaptically triggered inward current, *I*_CAN_ manifests postsynaptic burst-generating capacity. We ordered constituent neurons based on average *I*_CAN_ magnitude, which positively correlated with in-degree. Constituent large in-degree neurons have a greater probability of synaptic inputs arriving synchronously, compared with lower in-degree neurons, which maximizes *I*_CAN_ activation. In selective targeting experiments that preferentially ablated cells according to *I*_CAN_ rank-order, rhythm cessation occurred at lower tallies, which verifies the importance of *I*_CAN_ for rhythmogenesis in this model system.

Inspiratory burst generation relies on *I*_CAN_ in the Rubin-Hayes model (Rubin et al., 2009), as well as in other contemporary models (Toporikova and Butera, 2011; Jasinski et al., 2013) Experimental evidence shows that *I*_CAN_ is expressed in preBötC neurons and contributes substantially to bursts (Crowder et al., 2007; Pace et al., 2007b; Mironov, 2008, 2013; Pace and Del Negro, 2008; Rubin et al., 2009; Mironov and Skorova, 2011). Nevertheless, the importance of *I*_CAN_ and burst generation has recently been challenged by an alternative mechanism based on less intense “burstlets” that may reflect recurrent excitation at the network level in the absence of robust bursts and motor output (Kam et al., 2013a). Our modeling framework could be correct, or correct in part, about recurrent excitation and network dynamics, while wrongly asserting the central importance of *I*_CAN_, but that remains to be evaluated.

The other major cellular property that promotes network rhythmogenesis was gleaned from pre-inspiratory latency analysis. Constituent neurons with high input resistance (low *g*_leak_) are more sensitive to incoming synaptic inputs, which enhance graded potentials postsynaptically and lead to early spiking in the pre-inspiratory phase. Therefore, these high input resistance neurons play a more important role than those with low input resistance in the process of recurrent excitation. This conclusion has considerable experimental credibility (Del Negro et al., 2002a; Koizumi and Smith, 2008).

We rank-ordered the constituent neurons according to pre-inspiratory latency and plotted latency rank order versus cycle time (Fig. 8). The slope of this curve (its derivative) quantifies constituent neurons recruited per unit time, a measure of the rate of recurrent excitation (Fig. 9). Whether spontaneously active, or evoked to burst via a transient stimulus, if the rate of recurrent excitation does not achieve 1 neuron/ms, then a network-wide burst cannot occur. Therefore, rhythm cessation reflects not destruction of network topology or diminution of excitability, but rather impediments in the ability of excitation to spread through network constituents and achieve a threshold recruitment rate, which here measured 1 neuron/ms.

This threshold rate pertains to the model network assembled from Rubin–Hayes neurons according to parameters listed above (Materials and methods). The real system, or other models, may exhibit a slightly different threshold rate, but our analyses suggest that a particular threshold rate of recurrent excitation most likely exists and governs when spontaneous rhythms are sustainable in the preBötC whether studied *in vivo*, *in vitro*, or *in silico*.

*I*_Na-P_ is widely expressed in preBötC neurons. Here we set *g*_Na-P_ to 1 nS, which facilitates the richest set of dynamical behaviors in the Rubin–Hayes model (Dunmyre et al., 2011). *I*_Na-P_ is a predominantly somatic current that facilitates high frequency intra-burst spiking, which also underlies voltage-dependent bursting in preBötC neurons after synaptic isolation (Del Negro et al., 2002a,b,2005; Peña et al., 2004; Ptak et al., 2005). However, inspiratory synaptic drive appears to be largely mediated by convolved synaptic intrinsic currents and expressed on dendrites (Pace et al., 2007b; Mironov, 2008; Pace and Del Negro, 2008; Del Negro et al., 2011). Synaptic integration does not appear to be boosted by *I*_Na-P_ (Pace et al., 2007a; Koizumi and Smith, 2008). Elevating *g*_NaP,_ while balancing *E*_L_ to maintain slice-like cycle period in the network, did not systematically influence the ablation tally needed to cause rhythm cessation. Furthermore, *I*_Na-P_ active subnetworks did not degrade in response to cumulative ablations (Fig. 4*B*). Therefore, in Erdó́s-Rényi-structured preBötC-like networks assembled from Rubin–Hayes interneuron models, we conclude that *I*_Na-P_ does not play a specialized rhythmogenic role. Nevertheless, the role of *I*_Na-P_ continues to be investigated at different stages of development, in different physiological states (e.g., hypoxia and hypercapnia), and in different model organisms (e.g., rats, mice, and hamsters, among others).

### Disparities between experiments and simulations

In experiments we observed an exponential relaxation of the respiratory motor output amplitude in response to 10–15 cumulative Dbx1 neuron deletions (Wang et al., 2014). However, no such precipitous decrement in output amplitude occurred in simulations.

Experimentally the amplitude of respiratory motor output is monitored via hypoglossal (XII) nerve discharge (Funk and Greer, 2013). Dbx1 preBötC neurons in some cases project to the XII nucleus and thus serve in a premotor capacity (Wang et al., 2014). Deleting such neurons experimentally would presumably impair burst output amplitude without obligatory effects on burst frequency. In contrast, the amplitude in simulations is computed from the running-time spike histogram, which is based on the raster plot of preBötC rhythmogenic neurons. As explained earlier, *I*_CAN_ has a biphasic influence on action potential generation. Intra-burst spiking decreases when *I*_CAN_ is too low or too high. Therefore, as constituent neurons are deleted from the network and the overall *I*_CAN_ decreases, spike output capability is enhanced in some neurons, whereas in others that capability is diminished; the net result is that network-wide intra-burst spiking is maintained in the model system despite cumulative cellular ablations. This model does not account for the premotor population. Here, each model neuron contributes equally to the rhythmic amplitude. We contend that a more complete simulation of the respiratory brainstem network, which accounts for an intercalated premotor population of Dbx1 neurons will be necessary to fully replicate the experimental results, particularly the drop in amplitude in response to cumulative ablation (Wang et al., 2014).

Experimentally, 85 ± 20 constituent neuron deletions irreversibly terminated the respiratory rhythm (Wang et al., 2014), whereas the tally measured 39.1 ± 13.2 (mean ± SD) in simulations. What can explain this disparity?

We detected 705 Dbx1 neurons in the preBötC experimentally. Putative rhythmogenic neurons were identified by fluorescent protein expression despite the fact that Dbx1 derived neurons are not limited to the preBötC and that not all valid Dbx1 targets are respiratory (Bouvier et al., 2010; Gray et al., 2010; Picardo et al., 2013). In addition, a subset of Dbx1 preBötC neurons projects directly to the XII motor nucleus and thus may consist of premotor rather than rhythm-generating interneurons. Deleting these neurons is superfluous with regard to the tally of cellular ablations required to impair network functionality (Wang et al., 2014). By contrast, our simulations assume that all constituent neurons in the model network are rhythmogenic therefore removing any one of them can diminish network functionality. The biphasic effect of *I*_CAN_ on intra-burst spiking (see Results, Active subnetwork analysis) explains why the amplitude of inspiratory burst-related spiking does not decrease despite progressive decreases in the average magnitude of *I*_CAN_.

Another possible reason for the disparity in the ablation tallies may pertain to discrepancies in network complexity. The actual topology of the Dbx1 interneuron network in the preBötC remains unknown. Our model is reasonably well configured based on empirical data but it may lack clustering effects among constituent neurons that, in the real system, would endow greater robustness and increase the ablation tally needed stop the rhythm. We used Erdó́s-Rényi graphs for network structure because it is the most generalized random network model without any additional assumptions on the intrinsic positional differences among neurons, such as hubs or small-world properties. Thus, special network structures (e.g., hubs or small worlds) cannot explain rhythm cessation following cumulative cellular ablation.

Even though the experimental and simulation tallies are different, we contend that the model may provide insights into why the real preBötC core oscillator ceases spontaneous function when subjected to piecewise disassembly *in vivo* or *in vitro*. Cellular ablations do not destroy the structure of the underlying network (its constituent cells remain connected formally even after 30% of the network is removed), but rather hinder the rate at which neurons can recruit one another to start spiking in the inter-burst interval, i.e., the rate of recurrent excitation. For arrhythmic conditions, such as the *in vitro* model in its lesioned state, or the preBötC under pathological conditions *in vivo* (including saporin lesions or disease states leading to respiratory failure) our analyses suggest that restoration of network functionality may be possible if simultaneous activation of several units can be accomplished via exogenous stimulation or the strength of excitatory transmission among constituent neurons can be augmented.

## Synthesis

The decision was a result of the Reviewing Editor Muriel Thoby-Brisson and the peer reviewers coming together and discussing their recommendations until a consensus was reached. A fact-based synthesis statement explaining their decision and outlining what is needed to prepare a revision is listed below. The following reviewers agreed to reveal their identity: Nicholas Mellen, Jeffrey Smith

The authors' utilized E-R random networks to allow evaluation of network topology as assessed by various metrics of connectivity applied to the E-R network representation following simulated cumulative neuronal ablations. This allowed evaluation of the potential breakdown of network topology as a contributor to the disruption of rhythm generation, as distinguished from effects of cumulative ablation on cellular and synaptic factors contributing to network excitability. The paper includes analysis of the role of neurons with larger ICAN and the rate of neuronal activity recruitment by recurrent excitation on the capability of the model networks to generate population-wide bursts. The overall conclusion is that ablation-related termination of rhythm generation does not reflect a breakdown in network topology in the model (as concluded from previously published study) or reduction of excitability (e.g., shift in the mean level of neuronal depolarization), but can be attributed to failure of excitation to spread throughout the network to achieve a critical threshold recruitment rate related to neuronal spiking during the inter-burst interval. Two particularly interesting new results are that (1) cumulative deletions decrease the active sub-network size along with the instantaneous frequency in a step-like fashion associated with a shift in the periodic attractor of the system, and (2) selectively targeting neurons with high ICAN decreased the ablation tally required to stop the rhythm consistent with the hypothesis that these neurons might play a more important role in rhythmogenesis.

The results are an interesting extension of the previously published modeling data applying E-R random networks and the Rubin-Hayes cellular model, although it would be important to have a more explicit statement in the Discussion elaborating on the specific advance(s) made in these studies (e.g., novel detailed analysis of the impact of specific cellular and synaptic properties on the network-wide dynamics with cumulative cell ablations). The overall conclusions are intriguing, and the authors discuss appropriate caveats associated with the assumptions/limitations of their model and certain discrepancies between the simulation results and previously published laser ablation experimental results.

Both reviewers agree that the results presented here provide novel perspective/framework for possibly understanding how progressive loss of neural function in the respiratory network alters network dynamics and might impact inspiratory rhythm generation at least in the isolated rhythm generation kernel in vitro. Also they acknowledge that matching experiments with realistic simulations is a powerful heuristic that needs to be more broadly used. However they raised some concerns that should be appropriately addressed.

Specific suggestions for major revision:

1- The Authors need to provide a justification for why INaP wasn't included in the in silico experiment as a dependent variable, rather than as a constant. Because performing a full analysis of INaP would require extensive simulation probably not achievable in a decent timeline such additional experiments are not required but this point must definitively be discussed further. Some specific comments are as follows:

In the method section the authors write: "Each node (i.e., neuron) is populated with a Rubin-Hayes preBötC model (Rubin et al., 2009), which features Hodgkin-Huxley-like spiking currents and four additional currents: calcium-activated non-specific cation current (ICAN) (Crowder et al., 2007; Pace et al., 2007; Mironov, 2008, 2013; Pace and Del Negro, 2008; Mironov and Skorova, 2011), persistent sodium current (INaP) (Del Negro et al., 2002; Ptak et al., 2005; Koizumi and Smith, 2008)". It is unclear why the response of the network to deletions under conditions where INaP was varied was not also explored in their simulations. Because of its region of negative slope conductance, INaP confers nonlinear response properties to neurons, which could serve to amplify synaptic inputs. The Authors should at least provide a rationale for why INaP wasn't also assigned from a Gaussian distribution, and explored as a dependent variable that might influence network level bursting.

This issue comes up again at the end of Methods, where the Authors write: "Synaptic excitation evokes the inward current ICAN, which predominantly influences burst generation in the Rubin-Hayes model." The reader should be provided a rationale for this claim, rather than having to review the publication in which the Rubin-Hayes model was developed. In numerous publications, the presence of substantial numbers of preBötC neurons with INaP has been reported, and these conductances have long been proposed as playing a key role in rhythmogenesis. Further, the paucity of ICAN-based bursting neurons in P0-P5 rodents suggests that expression of ICAN is lower in the immediate post-natal period, which begs the question of how relevant the findings reported here are to the early post-natal slice. Finally, it is unclear why only the Rubin Hayes model of 2009 is considered here, when some authors proposed a model that integrates ICAN and INaP (Dunmyre, 2011). The Authors need to justify why interactions between ICAN and INaP were not explored in this simulation.

Also in highlighting the importance of pre-inspiratory latency (and hence low gleak), the Authors argue that a recurrent excitation threshold determines whether a network-wide burst occurs. Insofar as INaP functions as an amplifier of synaptic inputs (in that a small EPSP-induced depolarization can shift a neuron into the region of negative slope conductance), this parameter at a minimum should be included in discussion of factors contributing to network-wide recurrent excitation.

2- The Authors need to do a better job of disambiguating whether the model parameter ICAN is a network or cellular parameter, since ICAN magnitude is determined by the number of presynaptic inputs. As the paper is currently written, network and cellular properties appear to be conflated. If this is the case, this should be explicitly stated, since it is common in models to include currents, but these currents are usually not proportional to synaptic inputs or other network properties.

"Here we show that this loss of function is not linked to deterioration of network structure as determined by a subset of formal network measures. Furthermore selectively targeting neurons that have a large number of incoming synapses decreases the ablations tally considerably, emphasizing the importance of these properties for spontaneous rhythmic function." This claim looks incoherent, since "neurons that have a large number of incoming synapses" seems like a network measure (hub). In Figure 1, they show that during random ablation, network measures remain relatively constant, but they don't show how network measures are affected by targeted deletion of neurons with a large number of incoming synapses. Intuitively, networks measures would be sensitive to targeted deletion, which suggests that loss of function is sensitive to disruption of network structure, since targeted deletion of neurons with many synaptic inputs (i.e. large ICAN) leads to loss of rhythmicity after fewer deletions. In the experiments in which high ICAN neurons were serially deleted, leading to more rapid network silencing, do the Authors have a record of the connectivity between network constituents over successive deletions? If they do, the authors should add a figure showing how network measures are affected by targeted deletions. If targeted deletions are not accompanied by changes in network measures, then what they write stands, otherwise, the claim that network wide rhythmogenesis is insensitive to deterioration in network measures doesn't hold.

Also Figure 6 is confusing. In Methods (page 5), it appears that gCAN is initialized by selecting a value from a Gaussian distribution. Thereafter, in Results (page 13), the Authors write: "Because ICAN was formulated as a synaptically triggered inward current based on experimental evidence (Crowder et al., 2007; Pace et al., 2007; Mironov, 2008, 2013; Pace and Del Negro, 2008; Rubin et al., 2009; Mironov and Skorova, 2011), its magnitude reflects spiking in presynaptic partners (which drive synaptic activation of ICAN) yet is a good measure of postsynaptic burst-generating activity." Thus, it would appear that the value of ICAN can be considered a surrogate of presynaptic drive. In the context of Figure 6 they write: "ICAN generates inspiratory bursts in the Rubin-Hayes preBötC interneuron model. Therefore, we predicted that neurons with greater ICAN, which appear more frequently in the active sub-network, play a more important role in rhythmogenesis. We ordered the neurons according to average ICAN, which was correlated with in-degree (Figure 6A)." Because, by construction, ICAN magnitude reflects presynaptic drive (from page 13), it seems obvious that average ICAN would be correlated with in-degree, since ICAN magnitude "reflects spiking in presynaptic partners", and the number of presynaptic partners is what is captured by in-degree. If this is correct, then figure 6A is redundant. But again more problematic is the conflation of a network parameter (number of presynaptic partners) with a current parameter. The Authors want to argue for the importance of ICAN, but the way they have constructed the model, ICAN appears to conflate biophysics and connectivity. Because gsyn is scaled to the total number of inputs, only ICAN varies with presynaptic partners, yet it is not explicitly presented as a network parameter. Taken together, it is thus not surprising that deletion of neurons with large ICAN led to more rapid silencing of the network: the neurons with the most presynaptic partners were being deleted from the network, but this seems to be more due to a network property than a biophysical property. Thus, in stating: "These data indicate that the neurons with higher ICAN tend to play a more important role.", the focus appears to be on a conductance, but the conductance magnitude is determined by the number of presynaptic partners. The Authors need to do a better job teasing apart what ICAN quantifies.

In the same line, in the discussion the Authors state: "The model preBötC does not break down topologically in response to cumulative ablation of its constituent neurons, as we determined by a suite of global and local metrics." As suggested above it would be useful if these metrics were also computed for networks undergoing targeted deletion of high-ICAN neurons. Intuitively, it would be expected that the more rapid disruption of network-wide rhythm would be accompanied by topological breakdown, since high ICAN neurons are neurons with many synaptic inputs. This conjecture is corroborated in a later passage: "We ordered constituent neurons based on average ICAN magnitude, which positively correlated with in-degree." Since in-degree is a topological feature, the observation that deletion of high in-degree neurons hastens network quiescence is at odds with the claim that the model preBötC does not break down topologically.

3- One reviewer suggests that the authors show in a figure an example of the E-R random network (e.g., for one of the simulated sub-networks) to give the reader a clearer picture of the network structure simulated.

4- Abstract: "However, our analyses indicate that model preBötC networks remain topologically intact even after rhythm cessation, suggesting that network dynamics coupled with structural properties of underlying static graphs are responsible for rhythm cessation." The authors indicate that rhythm cessation can be explained by the "structural properties of underlying static graphs". Static graphs are never discussed in the manuscript. Assuming that at least some of the readers of this paper are biologists, it cannot be assumed that "static graphs" will be a familiar term with precise connotations. It is also problematic that the mechanism invoked to explain rhythm cessation in the Abstract is never referred to or elaborated on in the rest of the manuscript. The Authors should either elaborate in the Results/Discussion section on what they mean by the structural properties of underlying static graphs, or delete this phrase from the Abstract.

5- In the discussion of pre-inspiratory latency, the Authors write (page 22): "The other major cellular property that promotes network rhythmogenesis was gleaned from pre-inspiratory latency analysis. Constituent neurons with high input resistance (low gleak) are more sensitive to incoming synaptic inputs, which enhance graded potentials postsynaptically and lead to early spiking in the pre-inspiratory phase. Therefore, these high input resistance neurons play a more important role than those with low input resistance in the process of recurrent excitation." This conclusion has considerable experimental credibility (Del Negro et al., 2002; Koizumi and Smith, 2008).

Thus, pre-inspiratory latency is determined by model parameter gleak. Nowhere in the Results section is this strong dependence mentioned. The Authors need to revise the Results section to explicitly state that pre-inspiratory latency is determined by model parameter gleak.

6- The description of active subnetworks (page 13 onwards) is confusing. In the body of the manuscript, they write: "Figure 3A plots the size of the active sub-network for five different thresholds (θ: -2 pA, -3 pA -3.5 pA, -4 pA, and -5 pA) during the course of one representative simulation." But in the figure, thetas are denoted by subscript, so it is unclear which threshold is represented by which curve. This is not disambiguated in the legend, which refers to thetas by subscript only. In the body of the text, the mapping between subscript and threshold magnitude is identified, but current amplitude has to be calculated from the subscript and the increment size. This is needlessly confusing. Furthermore, why is the subnetwork size unitless? Aren't the units here the number of simulated neurons in the subnetwork? If this isn't the case, what is the subnetwork made up of, and why is it unitless? There may be a reason for this, but none is given.

7- In the Results section "Rate of recurrent excitation", the Authors write: "Whenever the rate of recurrent excitation exceeded 1 neuron/ms a network-wide burst occurred, even when the interburst interval was long. However, if the rate of recurrent excitation did not reach 1 neuron/ms, then no network-wide burst could be spontaneously generated (Figure 8a-d, which correspond to Figure 7a-d)." This observation is difficult to interpret. At one extreme it might be interpreted as a hard constraint on how sparse a network can get before synchronous activation becomes impossible. At the other extreme, it is an artifact of the model's temporal resolution (perhaps if the time-step were reduced to 0.1 s, a more dispersed recurrent excitation rate threshold would be found). Somewhere in the middle, this threshold is likely to be labile to biophysical parameters. The Authors need to provide the reader with an interpretation of this 1 neuron/ms threshold. Does it reflect a biological strength, or is it due to how the model was implemented (i.e. time-step size or other feature of the implementation)?

8- The description of how the network degrades with neuron deletion is confusing. In the Methods section, the Authors write: "Network models were subject to 100 random deletions because less than 100 Dbx1 neuron laser ablations was experimentally demonstrated to silence spontaneous respiratory rhythm in slices (Wang et al., 2014)." In Figure 1 and results, the effect of deletions is described in terms of percent of total deletions. Since up to 100 neurons were deleted, why not just label the abscissa as "number of neurons deleted"?

9- In the section "Disparities between experiments and simulations", the Authors write: "In experiments we observed an exponential relaxation of the respiratory motor output amplitude in response to 10-15 cumulative Dbx1 neuron deletions (Wang et al., 2014). However, no such precipitous decrement in output amplitude occurred in simulations. Experimentally the amplitude of respiratory motor output is monitored via hypoglossal (XII) nerve discharge (Funk and Greer, 2013b). Dbx1 preBötC neurons in some cases project to the XII nucleus and thus serve in a premotor capacity (Wang et al., 2014). Deleting such neurons experimentally would presumably impair burst output amplitude without obligatory effects on burst frequency. The amplitude in simulations is computed from the running time spike histogram, which is based on the raster plot of all preBötC rhythmogenic neurons. We have not accounted for the premotor population." This same issue is addressed in Results (page 14), but is ascribed to a completely different mechanism: "It may seem counterintuitive that the network burst output did not change during the ablation sequence (Figure 2, red traces) while the active sub-network size decreased stepwise (Figs.3-5). Although the average ICAN declined during the sequence, network burst output remained stable because ICAN has a biphasic influence on the ability to generate action potentials; intra-burst spiking decreases when ICAN is too low or too high. ICAN generally ensures that inspiratory bursts remain more robust and larger in magnitude than is needed to sustain rhythmogenesis (q.v., (Kam et al., 2013b). However, the property of ICAN to enhance burst magnitude causes depolarization block of spiking when its magnitude is large, which cuts down on the number of spikes per burst (Rubin et al., 2009) (also see example cells in Figure 2). Thus, as the average ICAN decreases during the cumulative cell ablation sequence, neurons with low gCAN decrease burst amplitude and generate fewer intra-burst spikes, whereas other neurons with larger gCAN generate more intra-burst spikes because the ability of ICAN to cause depolarization block of spiking is reduced during the course of the ablation sequence. As a result, the network burst output - as quantified by the running time spike histogram - does not decline." The Authors should rewrite this - either by moving all of the explanations to Discussion where they are presented in an integrated manner, or by eliminating one or the other.

10- In the Discussion the Authors write: "The animals developed pathological and ultimately fatal breathing phenotypes over the course of several days, during which time saporin killed off a significant fraction of the rhythmogenic preBötC core." This is inaccurate. In the cited publication, it was specifically stated that the animals survived following SP-SAP lesioning of NK1Rs:

When taken together with previous results1-3,10,12,13, our interpretation is that normal breathing in mammals requires an intact preBötC, with NK1R neurons having a necessary role. In their absence, an ataxic rhythm sufficient to maintain life can still be generated in conscious rats by undetermined structures, which may include non-NK1R expressing neurons in the pre-BötC or ventrolateral respiratory column14, other respiratory regions13, vestigial brainstem rhythmogenic networks15,16 or even structures normally underlying voluntary or emotional control of respiratory muscles.

11- The authors should explicitly outline in the Discussion how the present simulations results have importantly extended the previously published modeling results.

12- Suggestions for figures:

The Authors might be able to replace the 2 panels of figure 3 with a heat map, with burst index on the abscissa, thetas on the ordinate, and active subnetwork size described by the color of the heat map. The basic take-homes of these two figures (that for a given ICAN threshold, the active subnetwork size decreases in a step-wise manner as a function of deletions, that after a sufficient number of deletions, only the lowest level of ICAN activation persists) will be more obvious in the heat map.

Visual inspection of the time-series plots above each return map in figure 4 reveal that frequency and subnetwork size covary. This relationship would be made clearer by presenting a scatter plot of normalized frequency X subnetwork size: points will lie near the identity line, but will not be continuously distributed, because of the discontinuities seen in the Poincaré plots. This would obviate the need for figure 5, which makes essentially the same point.

Another confusing issue: why is subnetwork size now presented in number of neurons, whereas in the preceding figure, subnetwork size is unitless?

Minor comments:

- In specifying model parameters (page 6) the Authors give values for some parameters twice:

ᵅ4;can = 4 ± 0.75 ᵅB;ᵄ6;, ᵃ8;can = 0 ᵅA;ᵄ9;, ᵅ4;syn = 2.5 ᵅB;ᵄ6;, ᵃ8;syn = 0 ᵅA;ᵄ9;, ᵰ3;m = −36 ᵅA;ᵄ9;,

ᵅ4;can = 4 ᵅB;ᵄ6;, ᵃ8;can = 0 ᵅA;ᵄ9;, ᵅ4;syn = 3.25 ᵅB;ᵄ6;, ᵃ8;syn = 0 ᵅA;ᵄ9;, ᵰ3;m = −36 ᵅA;ᵄ9;,

The Authors need to either correct this error, or explain why two values are assigned to the same parameter.

- p. 13, para. 3. "...the size of the active sub-network for five different thresholds..." These threshold values of ICAN seem small. It would be helpful to provide some information on the maximum values of ICAN for the representative sub-network for comparison to the threshold values.

p. 16, para. 1, line 4. For readers unfamiliar with graph theory, define and elaborate on the significance of the in-degree measure used to correlate with ICAN ordering.
